# Peer review practices by medical imaging journals

**DOI:** 10.1186/s13244-020-00921-3

**Published:** 2020-11-27

**Authors:** Thomas C. Kwee, Hugo J. A. Adams, Robert M. Kwee

**Affiliations:** 1 Medical Imaging Center, Department of Radiology, University Medical Center Groningen, University of Groningen, Hanzeplein 1, P.O. Box 30.001, 9700 RB Groningen, The Netherlands; 2 Department of Radiology and Nuclear Medicine, Amsterdam University Medical Center, University of Amsterdam, Amsterdam, The Netherlands; 3 Department of Radiology, Zuyderland Medical Center, Heerlen, Sittard-Geleen, The Netherlands

**Keywords:** Bias, Journal article, Medical imaging, Peer review

## Abstract

**Objective:**

To investigate peer review practices by medical imaging journals.

**Methods:**

Journals in the category "radiology, nuclear medicine and medical imaging" of the 2018 Journal Citation Reports were included.

**Results:**

Of 119 included journals, 62 (52.1%) used single-blinded peer review, 49 (41.2%) used double-blinded peer review, two (1.7%) used open peer review and one (0.8%) used both single-blinded and double-blinded peer reviews, while the peer review model of five journals (4.2%) remained unclear. The use of single-blinded peer review was significantly associated with a journal’s impact factor (correlation coefficient of 0.218, *P* = 0.022). On subgroup analysis, only subspecialty medical imaging journals had a significant association between the use of single-blinded peer review and a journal’s impact factor (correlation coefficient of 0.354, *P* = 0.025). Forty-eight journals (40.3%) had a reviewer preference option, 48 journals (40.3%) did not have a reviewer recommendation option, and 23 journals (19.3%) obliged authors to indicate reviewers on their manuscript submission systems. Sixty-four journals (53.8%) did not provide an explicit option on their manuscript submission Web site to indicate nonpreferred reviewers, whereas 55 (46.2%) did. There were no significant associations between the option or obligation to indicate preferred or nonpreferred reviewers and a journal’s impact factor.

**Conclusion:**

Single-blinded peer review and the option or obligation to indicate preferred or nonpreferred reviewers are frequently employed by medical imaging journals. Single-blinded review is (weakly) associated with a higher impact factor, also for subspecialty journals. The option or obligation to indicate preferred or nonpreferred reviewers is evenly distributed among journals, regardless of impact factor.

## Key points

- Nearly all medical imaging journals use either a single-blinded peer review model (51.2%) or a double-blinded peer review model (41.2%).
- Reviewer preferences are optional by 40.3% and obligatory by 19.3% of medical imaging journals.
- There is a positive association between the use of a single-blinded peer review model and a journal’s impact factor (correlation coefficient of 0.218, *P* = 0.022), also for subspecialty journals (correlation coefficient of 0.354, *P* = 0.025).

## Background

Peer review refers to a formal system held by scientific journals, whereby a manuscript is scrutinized by persons who were not involved in its creation but are considered knowledgeable about the topic of the manuscript [1–3]. Peer review is considered of crucial importance for the selection and publication of quality science [1–3]. All medical imaging journals listed by the authoritative Journal Citation Reports [4] use peer review before manuscript publication. Unfortunately, the peer review process has some potential weaknesses which may undermine its effectiveness in ensuring the quality and fairness of published research [5]. Richard Smith, former editor-in-chief of the BMJ, once mentioned: “So peer review is a flawed process, full of easily identified defects with little evidence that it works. Nevertheless, it is likely to remain central to science and journals because there is no obvious alternative, and scientists and editors have a continuing belief in peer review. How odd that science should be rooted in belief” [6].

There are multiple peer review models. Single-blinded and double-blinded models are best known, but there are several other models including triple-blinded, quadruple-blinded, and open review systems [7, 8]. In single-blinded peer review, the reviewers know the identity of the authors but not vice versa [7]. In double-blinded peer review, the identities of both authors and reviewers are kept hidden from each other [7]. In the triple-blinded peer review model, the authors’ identity is also hidden from the handling editor during the submission process, and the quadruple-blinded peer review model is further augmented by hiding the identity of the handling editor [7]. Finally, in an open peer review model, both authors and reviewers know each other’s identity [7]. Each system has advantages and disadvantages [7]. Double-blinded and open peer reviews are most supported by the current literature [7]. The single-blinded peer review system has been shown to be susceptible to bias [7, 9–11].

Another important issue that may affect the validity of the peer review process is the recommendation of potential reviewers by the submitting authors upon manuscript submission [12]. In 2014, it became apparent that these practices are vulnerable to exploitation and hacking, because some authors performed “peer reviews” of their own manuscripts by using fabricated e-mail accounts [12]. In the aftermath of the scandals involving fake peer reviewers, many journals decided to turn off the reviewer recommendation option [12].

Currently, there is a lack of knowledge on the peer review practices of medical imaging journals. More insight into the integrity and fairness of the peer review process is required in order to better appraise the quality of published research and to identify potential targets for improvement. This information is relevant to the readership of any medical imaging journal (even for journals which hold a high standard), because all journals publish articles that refer to some degree to studies that have been published elsewhere. The currently available evidence is supportive of double-blinded or open peer review rather than single-blinded peer review [7, 9–11] and discourages the use of the reviewer recommendation option for authors [12]. It is therefore hypothesized that most medical imaging journals employ such practices and that such a trend is particularly seen for journals with a higher impact factor. Therefore, the purpose of our study was to investigate peer review practices by medical imaging journals.

## Materials and methods

### Study design

Our study used data available in the public domain and did not concern medical scientific research in which participants or animals were subjected to procedures or were observed. Therefore, it did not require institutional review board approval or informed consent. All 129 journals listed by the 2018 Journal Citation Reports in the category “radiology, nuclear medicine and medical imaging” as of April 2020 were eligible for inclusion [4]. Journals that allowed submissions by invitation only were excluded.

### Data collection

The editorial procedure on each journal’s Web site was carefully studied for the peer review model employed by the journal (i.e., single-blinded, double-blinded, triple-blinded, quadruple-blinded, open peer review, or other). If this information was not provided on the journal’s Web site, editors-in-chief or editorial managers were contacted to require information about the peer review model. In the case of no reply within two weeks, editors-in-chief and editorial managers were contacted again in a final attempt to retrieve this information. Furthermore, the manuscript submission system of each journal was accessed to determine the presence of an optional or obligatory reviewer recommendation, and the presence of an option to indicate nonpreferred reviewers. Finally, the impact factor of each journal was determined based on the information provided by the Journal Citation Reports as of April 2020 [4]. All data were collected by a single author (T.C.K.).

### Data analysis

The proportions of journals with single-blinded, double-blinded, triple-blinded, quadruple-blinded, open review, and other models were determined. The proportions of journals with optional or mandatory reviewer recommendations, and those with the option to indicate nonpreferred reviewers, were also assessed. Point-biserial correlation analyses were performed to determine the associations between the peer review model employed by the journal and the journal’s impact factor, between the presence of a reviewer recommendation option or obligation and a journal’s impact factor, and between the presence of an option to indicate nonpreferred reviewers and a journal’s impact factor. A subgroup analysis was performed for all medical imaging journals except radiotherapy journals, journals for physicists, engineers, and chemists, and journals related to a single country. Additional subgroup analyses were performed for general and subspecialty medical imaging journals separately, and for imaging journals with more and less than 1000 published articles per 2-year period separately. *p* values < 0.05 were considered statistically significant. Statistical analyses were executed using IBM Statistical Package for the Social Sciences (SPSS) version 26 (SPSS, Chicago, IL, USA).

## Results

### Medical imaging journals

Of the 129 journals listed by the Journal Citation Reports in the category “radiology, nuclear medicine and medical imaging,” ten were excluded because they allowed submissions by invitation only. The 119 journals that were included in our analyses had a mean impact factor of 2.205 (range: 0.413–10.975).

### Peer review models

**Fig. 1:**
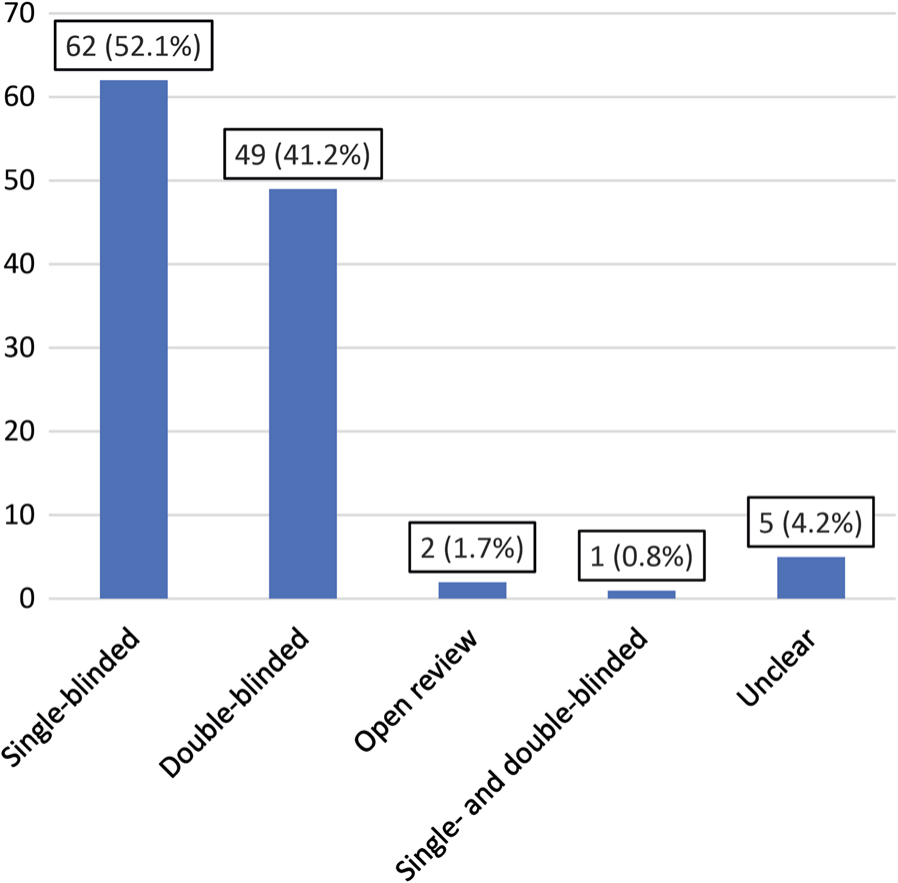
Peer review models used by 119 medical imaging journals (absolute numbers of journals with percentages between parentheses)

**Fig. 2:**
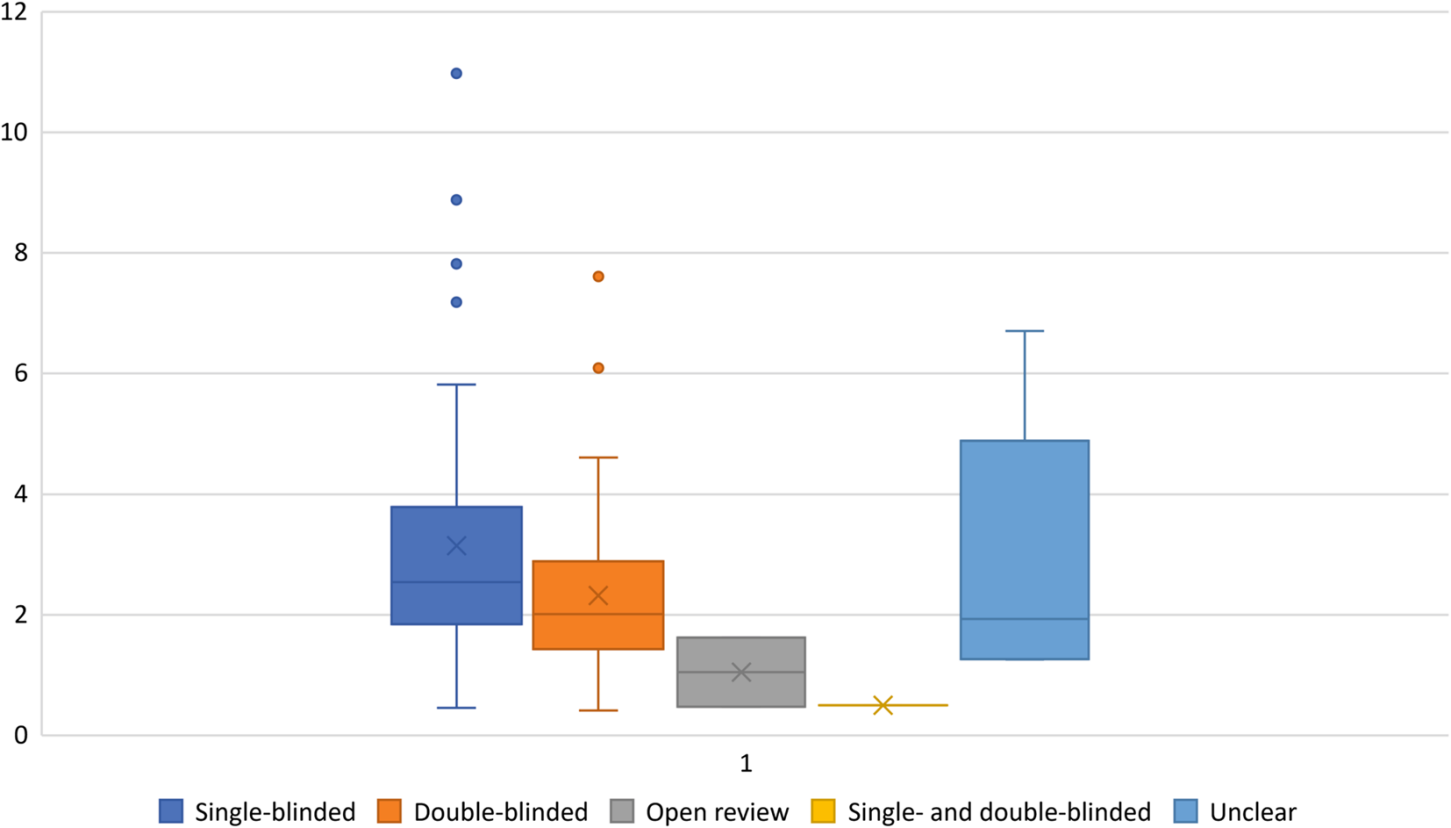
Box-and-Whisker plot show median (cross), quartiles (top and bottom lines of box), upper extreme value (upper whisker), lower extreme value (lower whisker), and outliers (circles) for journal’s impact factor according to peer review model

Of all 119 journals that were included, 62 (52.1%) used a single-blinded peer review model, 49 (41.2%) used a double-blinded peer review model, two (1.7%) used an open peer review model, and one (0.8%) used both a single-blinded and a double-blinded peer review model (depending on whether or not the submitting author disclosed the authors’ names on the first page of the manuscript), whereas for five journals the peer review model remained unclear (Fig. 1). There were no journals which used another type of peer review model. Seventy-two (60.5%) journals mentioned their peer review model on their Web site. A Box-and-Whisker plot of journal impact factor according to peer review model is shown in Fig. 2. Because nearly all journals used either the single- or double-blinded peer review model (97.4%), the correlation analysis was only performed for the single- and double-blinded models vs. journal impact factor. A point-biserial correlation coefficient of 0.218 (*P* = 0.022) indicated a positive association between the use of a single-blinded peer review system and a journal’s impact factor. On subgroup analysis, only subspecialty medical imaging journals had a significant association between the use of a single-blinded peer review system and a journal’s impact factor (point-biserial correlation coefficient of 0.354, *P* = 0.025) (Table 1).

**Table 1:** Subgroup analyses on the associations between the use of a single-blinded peer review system and a journal’s impact factor, between the presence of a reviewer recommendation option or obligation and a journal’s impact factor, and between the presence of a nonpreferred reviewer option and a journal’s impact factor

	Point-biserial correlation coefficient for the use of a single-blinded peer review system vs. a journal’s impact factor (*p* value)	Point-biserial correlation coefficient for the presence of a reviewer recommendation option or obligation vs. a journal’s impact factor (*p* value)	Point-biserial correlation coefficient for the presence of a nonpreferred reviewer option vs. a journal’s impact factor (*p* value)
All medical imaging journals (n = 119)	0.218 (*p = 0.022*)^a^	0.032 (*p* = 0.727)	0.064 (*p* = 0.492)
All medical imaging journals except radiotherapy journals, journals for physicists, engineers, and chemists, and journals related to a single country^1^ (n = 70)	0.220 (*p* = 0.777)^b^	− 0.019 (*p* = 0.878)	0.036 (*p* = 0.770)
Only general medical imaging journals (n = 27)^2^	0.053 (*p *= 0.810)^c^	0.083 (*p* = 0.681)	0.249 (*p * = 0.211)
Only subspecialty medical imaging journals (n = 41)^3^	0.354 (*p = 0.025*)^d^	− 0.102 (*p* = 0.527)	− 0.114 (*p* = 0.478)
Medical imaging journals with more than 1000 published articles per 2-year period (n = 36)^4^	0.286 (*p* = 0.964)^e^	0.130 (*p* = 0.448)	0.187 (*p* = 0.275)
Medical imaging journals with less than 1000 published articles per 2-year period (n = 34)^4^	0.180 (*p* = 0.341)^f^	− 0.179 (*p* = 0.310)	− 0.124 (*p* = 0.486)

*p* value < 0.05 were considered statistically significant (italics)

^1^Journals with a title that refers to a single country

^2^Excluding radiotherapy journals, journals for physicists, engineers, and chemists, journals related to a single country, and other journals that could not be classified as a general imaging journal

^3^Excluding radiotherapy journals, journals for physicists, engineers, and chemists, journals related to a single country, and other journals that could not be classified as a subspecialty journal

^4^Excluding radiotherapy journals, journals for physicists, engineers, and chemists, and journals related to a single country

^a^Five journals were excluded from this analysis because their peer review model remained unclear, and one journal was excluded from this analysis because it used both a single-blinded and a double-blinded peer review model

^b^Four journals were excluded from this analysis because their peer review model remained unclear, and one journal was excluded from this analysis because it used both a single-blinded and a double-blinded peer review model

^c^Three journals were excluded from this analysis because their peer review model remained unclear, and one journal was excluded from this analysis because it used both a single-blinded and a double-blinded peer review model

^d^One journal was excluded from this analysis because its peer review model remained unclear

^e^One journal was excluded from this analysis because it used both a single-blinded and a double-blinded peer review model

^f^Four journals were excluded from this analysis because their peer review model remained unclear

### Reviewer preferences

**Fig. 3:**
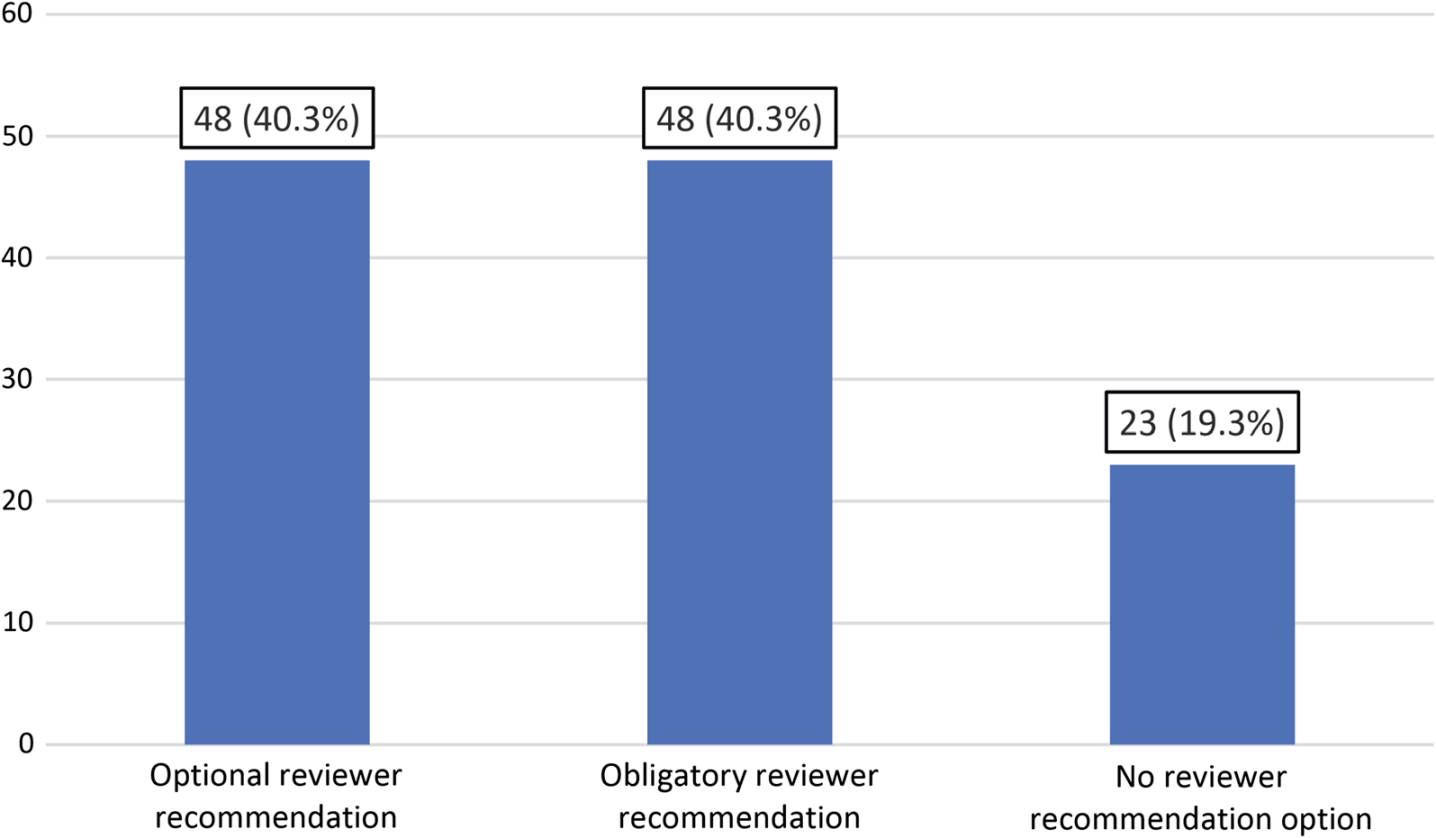
Reviewer recommendation options by 119 medical imaging journals (absolute numbers with percentages between parentheses)

Of all 119 journals that were included, 48 (40.3%) provided authors the option to indicate reviewer recommendations, 48 (40.3%) did not have a reviewer recommendation option, and 23 (19.3%) obliged authors to indicate reviewers on their manuscript submission systems (Fig. 3). The 23 journals with an obligatory reviewer recommendation required the suggestion of at least one reviewer (four journals), two reviewers (five journals), three reviewers (11 journals), four reviewers (one journal), and five reviewers (two journals). A point-biserial correlation coefficient of 0.032 (*P* = 0.727) indicated no significant association between the presence of a reviewer recommendation option or obligation and a journal’s impact factor. There were no significant associations on additional subgroup analyses (Table 1).

**Fig. 4:**
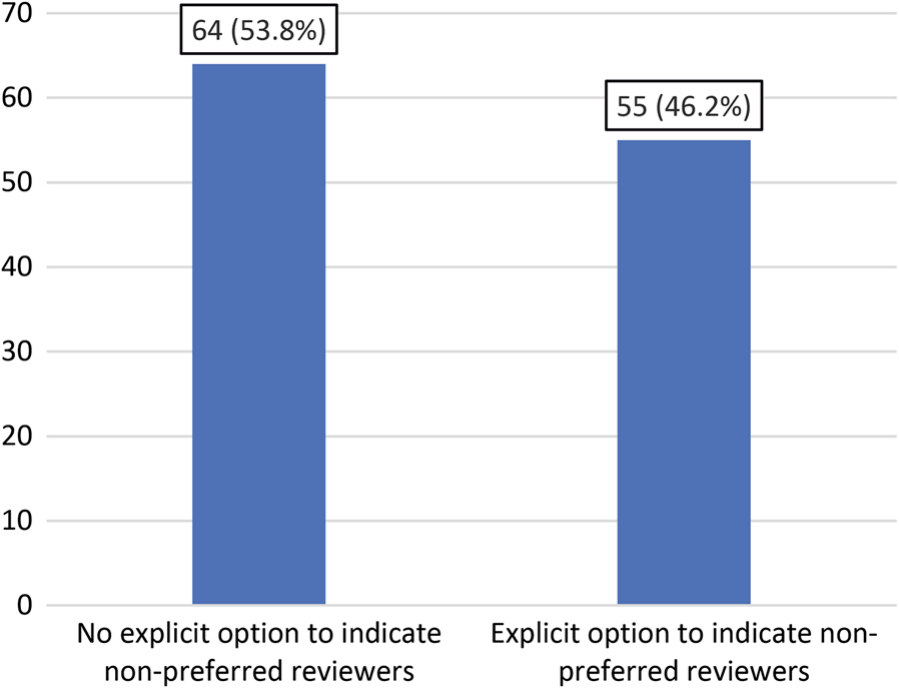
Option to indicate nonpreferred reviewers by 119 medical imaging journals (absolute numbers with percentages between parentheses)

Of all 119 journals that were included, 64 (53.8%) did not provide an explicit option on their manuscript submission Web site to indicate nonpreferred reviewers, whereas 55 (46.2%) did (Fig. 4). Fifty-three journals with a nonpreferred reviewer option did not indicate any limit for the number of nonpreferred reviewers, whereas two journals indicated that a maximum of five nonpreferred reviewers could be listed. A point-biserial correlation coefficient of 0.064 (*P* = 0.492) indicated no significant association between the presence of a nonpreferred reviewer option and a journal’s impact factor. There were no significant associations on additional subgroup analyses (Table 1).

## Discussion

Our study shows that the majority of medical imaging journals employ a single-blinded peer review model (52.1%), followed by a double-blinded peer review model (41.2%). However, there is ample evidence that the single-blinded peer review system is prone to bias [7, 9–11]. For example, it has been reported that reviewers are more likely to give higher manuscript ratings and recommend acceptance when prestigious authors’ names and institutions are visible than when they are not [9], that single-blinded reviewers are significantly more likely than their double-blinded counterparts to recommend papers from famous authors, top universities, and top companies for acceptance [10], and that single-blinded peer reviews may suffer from gender bias against women [11]. In addition, reviewers’ knowledge of the authors’ identities may render the review process susceptible to fraud when a conflict of interest exists between the authors and the reviewers. Therefore, it is worrisome that the single-blinded peer review model is employed by most medical imaging journals. Our results also indicate a weak but significant trend that the single-blinded peer review model is more frequently used by journals with a higher impact factor. Therefore, the concerns related to single-blinded peer review are certainly not only applicable to lower-ranked medical imaging journals. Interestingly, subgroup analyses showed that the association between single-blinded peer review and the journal’s impact factor was highest for subspecialty journals. The reason for the association between the use of a single-blinded peer review system and a journal’s impact factor remains unclear. However, it can be speculated that some journals use a single-blinded peer review system for reviewers to be able to check the credentials of the authors. Papers from authors with a prestigious track record are likely to receive a more favorable review which will increase the likelihood of (eventual) acceptation by the handling editor. In turn, published papers from authors with a prestigious track record are probably cited more frequently. This phenomenon can be referred to as the Matthew effect: “To those who have, shall be given; to those who have not shall be taken away even the little that they have” [6, 13]. Only two journals, with impact factors of 1.622 and 0.478, used an open peer review system. Other peer review systems, including triple- and quadruple-blinded systems, were not used by any of the journals. This is probably related to widespread long-term habituation to the use of single- and double-blinded systems, and more complexity and costs associated with the use of triple- and quadruple-blinded systems. This indicates that handling editors of all medical imaging journals are currently not blinded to the identity of the authors. However, many journals reject submissions without review, and although some experienced handling editors may have the expertise to make justified “direct reject” decisions, the possibility exists that they are prone to the same type of peer review bias that has been shown to exist for reviewers [7–11]. Even well-intentioned editors may be subject to unconscious bias, just as reviewers are [8]. It was also interesting to note that only a small majority of journals (60.5%) mentioned their peer review model on their Web site. The reason for this finding remains unclear, but it can be speculated that it is simply a neglected topic. This issue is another target for improvement, since transparency can be considered as one of the key components of scientific integrity.

Another important finding of our study is that there were just as many journals with and without the option to indicate reviewer preferences (both 40.3%), whereas the remaining journals (19.3%) obliged submitting authors to provide potential reviewers. This may also be considered worrisome, because recommendation of potential reviewers by the submitting authors has been shown to be vulnerable to exploitation, hacking, and peer review bias [12]. Furthermore, the obligatory reviewer recommendation is a potentially ethically compromising situation and a violation of author’s rights, because it forces authors to interfere with the review process [13]. The presence of a reviewer recommendation option or obligation on a journal’s manuscript submission system was independent of a journal’s impact factor, which indicates that this issue plays a role across the entire range of medical imaging journals. Although selecting appropriate reviewers costs time, an unbiased selection of potential reviewers is essential. Another potential reason for journals to employ the reviewer recommendation option or obligation is that they do not possess a large database of potential reviewers. The use of reviewer finding software could be a solution for these journals [14]. Yet another possibility is that recommendations for reviewers may also aid the handling editors' job enabling a faster turnaround time which may in itself improve the impact factor of a journal, although this remains an issue of speculation.

A nonpreferred reviewer option was present in nearly half (46.2%) of the included journals and was not associated with a journal’s impact factor. It is currently unclear how a nonpreferred reviewer option affects peer review. It may avoid peer review bias when authors disclose individuals with whom a conflict of interest exists. However, if authors indicate their wish to exclude certain knowledgeable individuals with stringent standards from whom they expect to receive a critical review which could lead to rejection, and the journal follows this request, the review process may potentially be biased in favor of the authors’ manuscript [15]. Further research is necessary to elucidate this element of the peer review process.

To our knowledge, there have been no previously published, similar studies on peer review practices by medical imaging journals. Nevertheless, the topic of peer review is regularly discussed [16–19] and two previous studies have investigated the efficacy of reviewer blinding in imaging journals [20, 21]. In a study by Katz et al. [20] that was published in 2002, original manuscripts submitted to two general radiology journals with double-blinded peer review policies during a 6-month period were reviewed. They found that 34% of submitted manuscripts contained information that potentially or definitely unblinded the identities of the authors or their institutions [20]. The most frequent unblinding violations were statement of the authors' initials within the manuscript, referencing work "in press,” identifying references as the authors' previous work, and revealing the identity of the institution in the figures. In a more recent study by O'Connor et al. [21], all reviewers of manuscripts submitted to the American Journal of Neuroradiology from January through June 2015 were surveyed in order to assess whether they were familiar with the research or had knowledge of the authors or institutions from which the work originated. Their survey revealed that reviewers correctly identified the authors in 90.3% of cases and correctly stated the institutions in 86.8% of cases [21]. Unblinding resulted from self-citation in 34.1% for both authorship and institutions [21]. Unsurprisingly, author familiarity and institution familiarity were significantly associated with greater manuscript acceptance (*P* < 0.038 and *P* < 0.039, respectively) [21]. The studies by Katz et al. [20] and O'Connor et al. [21] underline the responsibility of both authors and journals in ensuring that manuscripts are adequately blinded before sent out for peer review [22].

Our study had some limitations. First, it did not compare the validity of different peer review models. A randomized trial has yet to be performed to investigate whether any peer review model is more prone to bias in the medical imaging field. However, the current literature favors double-blinded and open peer reviews over single-blinded peer review models [7, 9–11]. There is no reason to assume why this concept would be different for medical imaging journals. In addition, empirical evidence has already shown the danger of using a reviewer recommendation option on a manuscript submission system [12]. Second, our study did not assess for any temporal changes in peer review practices. As such, it remains unclear whether peer review standards in the medical imaging field have improved according to the above-mentioned insights that have appeared in the recent literature [7, 12]. Nevertheless, our study sets a benchmark which could be used to monitor and to possibly improve upon in the future. Third, metrics of peer review practices were correlated with journal impact factors. However, the impact factor can be influenced and biased by many factors, and extension of the impact factor to the assessment of journal quality may be inappropriate [23]. Fourth, we did not compare peer review practices of journals in the medical imaging field to journals in other areas, because this was beyond the scope of the present study.

In conclusion, single-blinded peer review and the option or obligation to indicate preferred or nonpreferred reviewers are frequently employed by medical imaging journals. Single-blinded review is (weakly) associated with a higher impact factor, also for subspecialty journals. The option or obligation to indicate preferred or nonpreferred reviewers is evenly distributed among journals, regardless of impact factor.

## Data Availability

Data and materials are available upon request to the authors.

## Abbreviation

SPSS: Statistical Package for the Social Sciences
